# Knowledge, Attitudes, Risk Perceptions, and Practices of Spanish Adolescents Toward the COVID-19 Pandemic: Validation and Results of the Spanish Version of the Questionnaire

**DOI:** 10.3389/fpsyg.2021.804531

**Published:** 2022-01-04

**Authors:** Alejandra Aguilar-Latorre, Ángela Asensio-Martínez, Olga García-Sanz, Bárbara Oliván-Blázquez

**Affiliations:** ^1^Institute for Health Research Aragón (IIS Aragón), Zaragoza, Spain; ^2^Primary Care Prevention and Health Promotion Network (redIAPP), Madrid, Spain; ^3^Department of Psychology and Sociology, University of Zaragoza, Zaragoza, Spain; ^4^Institute of Secondary Education San Miguel, Ministry of Education, Universities, Culture and Sports of the Government of the Canary Islands, Tenerife, Spain

**Keywords:** adolescents, COVID-19, validation, knowledge, attitudes, risk perceptions, practices

## Abstract

**Background:** Adolescence is a period with physical, psychological, biological, intellectual, and social changes in which there is usually little perception of risk. COVID-19 has generated constant situations of change and uncertainty worldwide. During the pandemic, the acquisition of preventive behaviors has been relevant. Various studies carried out with adults associate risk perception and the implementation of preventive behaviors with knowledge about the COVID-19 and with age, but there are not many studies with adolescents. Therefore, the objective is to validate, in Spanish, the questionnaire of the knowledge, attitudes, risk perceptions, and practices of adolescents toward the pandemic, and analyze it according to sociodemographic characteristics.

**Method:** This study was a descriptive cross-sectional study, which included adolescents between the ages of 12–18 (*n* = 354). First, a translation and a back-translation of the questionnaire were performed. The questionnaire was presented in several high schools chosen by convenience sampling and following a non-probabilistic snowball sampling. Reliability and validity analyses were then carried out and the relationships between the different sociodemographic variables (gender, place of residence, level of education, if the person was in a sentimental relationship, and financial aid) were analyzed.

**Results:** The reliability of the questionnaire is acceptable (ordinal alpha = 77%). Knowledge was higher in women, and in those with a higher level of education; and were lower in those who lived in smaller towns, as well as in those who had a member of their family receiving financial aid. In terms of attitudes and risk perceptions, younger adolescents had higher scores, and those who had a member of their family receiving financial aid, lower.

**Conclusion:** The questionnaire is a reliable tool in the Spanish adolescent population. Knowledge was influenced by gender, place of residence, level of education, and financial aid. Attitudes and risk perceptions were influenced by age and financial aid. For practices, no predictors were found. In general, adolescents scored lower on knowledge about COVID-19, but they scored higher on COVID-19 safety practices.

## Introduction

Biological hazards, such as infectious disease outbreaks, are considered one of the main health risks for the human population (Chan et al., 2020). In March 2020, the World Health Organization (WHO) determined that COVID-19 (SARS-CoV-2) was a pandemic and the whole world has been awaiting its evolution since then. In August 2021, Spain had a total of 4,566,571 cases with a diagnosis of COVID-19 and 81,931 deaths from COVID-19 (Secretaría General de Sanidad, 2021). The highest proportion of COVID-19 cases occur in the 15–59 age group (66% of the total), with the 15–29 age group being the most represented and consisting of 19.7% of the cases, followed by the 40–49 age group, representing 17.0% (Equipo Covid-19. RENAVE. CNE. CNM (ISCIII), 2021).

Numerous studies try to measure the different variables that influence the level of compliance with the preventive health measures imposed and/or recommended to the population. The WHO itself warns that the best way to prevent and slow down transmission is to be well informed about the COVID-19 virus, the disease it causes, and how it spreads (World Health Organization, 2021). Some variables such as being a man, having a lower level of education, belonging to a disadvantaged social group, and/or being elderly have been shown to be related to a lower level of knowledge and poorer preventive practices (Honarvar et al., 2020). In the same regard, a recent national survey research conducted in the United States found that women had more knowledge and better attitudes regarding preventative recommendations, whereas individuals with lower education and income had less knowledge (Mondal et al., 2021).

Risk perception has been shown to influence health-related behaviors and change risk behaviors (Glanz et al., 2008). Risk perception has been defined as the subjective judgment that people make regarding the characteristics, severity, and way in which risk is managed (Cori et al., 2020). Risk perception presents significant associations with risk communication and the adoption by the population of self-protection measures (de Bruin and Bennett, 2020; Duan et al., 2020). In this way, understanding the population’s risk perception is an effective strategy for governments that seek to encourage their citizens to adopt self-protection measures during the COVID-19 pandemic. Until the majority of the population receives an effective vaccine, or until there is a definitive treatment, preventive behaviors are the only way to overcome the disease/pandemic (Shahnazi et al., 2020). Therefore, it is essential to continue working in order to develop tools that allow the design of effective prevention strategies.

Various studies carried out in adults associate risk perception and the implementation of preventive behaviors with knowledge about the COVID-19 pandemic and with age (de Bruin and Bennett, 2020; Honarvar et al., 2020; Olapegba et al., 2020). Likewise, studies reflect gender differences regarding the perception of risk in adult men and women, with men presenting the most reckless practices (Honarvar et al., 2020; Iorfa et al., 2020; Olapegba et al., 2020).

Currently, there is limited scientific evidence available to understand the patterns of knowledge, perception, attitude, and behavior undertaken by the population (Chan et al., 2020). Risk situations in adolescents are related to a complex psychosocial interaction that promotes them. Therefore, it is crucial to examine not just the consequences of the behaviors, but also their antecedents and the potential roles that these behaviors may play in their lives (Peñaherrera Sánchez, 1998). Adolescence is the stage of an individual’s life between childhood and adulthood. According to the WHO, it is a period of human growth and development that happens between the ages of 10 and 19, with there being physical, psychological, biological, intellectual, and social changes that end when adulthood is reached. There is currently a very small number of studies that use adolescents to analyze the variables which influence their protective behaviors. However, some studies have noted the existence of a significant positive correlation between these behaviors and variables such as self-efficacy (Fathian-Dastgerdi et al., 2021). Some studies also show differences in age ranges in terms of risk perception against COVID-19; however, most studies analyzing risk perception have been carried out in the adult population; over 18 years of age (Iorfa et al., 2020; Serwaa et al., 2020; Monteiro et al., 2021).

In order to determine peoples’ knowledge, attitudes, risk perceptions, and practices, Honarvar et al. (2020) designed the knowledge, attitudes, risk perceptions, and practices of adults toward COVID-19 questionnaire. The purpose of this questionnaire is to provide policymakers pieces of field-based evidence to help them in the management of the COVID-19 pandemic. The instrument was validated in the adult Iranian population and consisted of 52 questions: 6 demographic items; 17 knowledge-based and 18 practice-based questions about transmission, prevention, and the treatment of COVID-19; 10 questions regarding attitudes and risk perceptions; and 1 question about the source of COVID-19 news. The questionnaire has not yet been validated in any other country, or in any other type of population.

This research arises from the need for a measurement instrument that allows for the estimation of the knowledge, attitudes, risk perceptions, and practices of adolescents toward the COVID-19 pandemic. Therefore, the objective of this study is to validate a pre-existing questionnaire in Spanish adolescents and analyze it according to sociodemographic characteristics.

## Materials and Methods

### Design

First, we translated the original questionnaire (into Spanish) and back-translated it (into English). Then, we validated the original knowledge, attitudes, risk perceptions, and practices of adults toward COVID-19 questionnaire (Honarvar et al., 2020), in Spanish adolescents.

### Instruments

The instrument used was the questionnaire developed by Honarvar et al. (2020) entitled: knowledge, attitudes, risk perceptions, and practices of adults toward COVID-19 questionnaire. To score the knowledge, eight questions included three answer options (completely correct, incompletely correct, and incorrect/not knowing the answer), while nine questions included two options (correct and incorrect/not knowing the answer). To score the practices, 13 questions were scored with a 5-point Likert scale and 5 questions were scored using a dichotomous scale (answers yes/no). The total score for knowledge was up to 25 points and for practices was 75. If the score obtained in the total achievable scores in the knowledge and practice sections was less than 40%, the result was considered “inappropriate.” If the result was 40–70%, it was considered “more or less appropriate.” If the result was greater than 70%, it was considered “appropriate.” The original questionnaire showed the reliability of the questionnaire with Cronbach’s alpha level of 64.1% for the knowledge subscale, 78.1% for the attitudes subscale, 82.3% for the practices subscale, and 65.5% for the entire questionnaire (Honarvar et al., 2020).

We also collected sociodemographic variables (gender, place of residence, level of education, if the person was in a sentimental relationship, and financial aid) through an *ad hoc* questionnaire.

### Participants and Sample Size

The study was carried out in a representative sample of Spanish population aged between 12 and 18 years. According to the INE (Instituto Nacional de Estadística [INE], 2019), there are a total of 4,813,817 inhabitants aged between 10 and 19 years old. With a margin of error of 5%, a probability of success of 95%, a confidence level of 95%, a precision level of 3%, and an estimated withdrawal rate of 20%, the sample size required was approximately 253 adolescents. The sample finally reached was 354, exceeding the needed sample size. The calculation was made with the free software Epidat version 4.2 (Consellería de Sanidade – Xunta de Galicia, 2016). The questionnaire was presented in several high schools, mainly situated in the communities of Zaragoza and the Canary Islands (Spain). The high schools were chosen by convenience sampling (i.e., the high schools where we had easy access and where we were able to make a first dissemination of the study were chosen) (Galloway, 2005). The sampling technique was non-probabilistic snowball sampling (i.e., research participants were asked to encourage others to answer) (Johnson, 2005). The questionnaire was also disseminated *via* social networks (Facebook,^1^ Instagram,^2^ and Twitter^3^), as more than 90% of the adolescent use them to communicate (American Academy of Child and Adolescent Psychiatry, 2018); and, in this way, we could reach a bigger sample with different characteristics.

### Procedure

First, a translation into Spanish and back-translation of the original questionnaire by Honarvar et al. (2020) were carried out by different translators. Then, an online survey through the Google Forms platform was created (see Supplementary Material). This was available through an access link, from October 2020 to January 2021. Participants accessed the survey through email, social networks, and *via* its dissemination by collaborating entities and institutes. The data was anonymized. Adolescents under 13 years of age needed the express consent of a parent or legal guardian.

### Data Analysis

First, a descriptive analysis of the characteristics of the study population and all the variables was carried out (percentages for categorical variables, and means and SD for quantitative variables). The face and content validity of the original questionnaire (Honarvar et al., 2020) were confirmed by an expert panel composed of experienced epidemiologists, virologists, and regional health legislators in three sessions. For the internal consistency analysis, ordinal alpha values were obtained (Contreras Espinoza and Novoa-Muñoz, 2018). For the construct validity analysis, a Confirmatory Factor Analysis (CFA) was performed (Brown, 2015). This type of analysis was conditioned by obtaining significant values of the Kaiser-Meyer-Olkin (KMO) sample adequacy index and the Bartlett sphericity test (*p* < 0.05) (Field, 2017). The correlation between knowledge and practices was evaluated using the Pearson correlation coefficient test. Univariate analysis was carried out by means of a *t*-test and an analysis of variance (ANOVA). A multivariate analysis was performed using linear regression checking collinearity and VIF factors. Participants with missing values were eliminated following the Listwise Deletion method (Allison, 2009). Data collection and statistical analyses were performed using Excel software, SPSS software (version 25.0) (IBM Corp, 2017), and the R statistical software environment (version 3.6.2) (R Core Team, 2019). All significant levels were established at *p* < 0.05.

## Results

A total of 354 adolescents participated in this study. Their mean age was 15.37 ± 1.55 years, with 65.3% being female (Table 1). Age was categorized into three groups: early, middle, and late (Hardin et al., 2017).

**TABLE 1:** Demographic characteristics of the sample.

Variables	Participants (*n* = 354)
**Age**	15.37 (±1.55)
Early (up to 14 y/o)	101 (28.5%)
Middle (between 15 and 17 y/o)	236 (66.7%)
Late (18 or more y/o)	17 (4.8%)
**Gender**	
Male	119 (33.6%)
Female	231 (65.3%)
Other	4 (1.1%)
**Birth country**	
Spain	329 (92.9%)
Others	25 (7.1%)
**Place of residence**	
City (more than 10,000 inhabitants)	232 (65.5%)
Town (less than 10,000 inhabitants)	122 (34.5%)
**What level of education have you completed?**	
None	2 (0.6%)
Primary school (6–12 y/o)	163 (46%)
Secondary school (12–16 y/o)	165 (46.6%)
Higher secondary (16–18 y/o)	20 (5.6%)
DK/NA	4 (1.1%)
**Are you currently in a sentimental relationship?**	
No	310 (87.6%)
Yes	44 (12.4%)
**Are any of your relatives you live with currently receiving financial aid?**	
Yes	48 (13.6%)
No	267 (75.4%)
DK/NA	39 (11%)

*y/o, years old; DK/NA, do not know/no answer.*

Regarding the internal consistency analysis, ordinal alpha values were obtained. Firstly, regarding all the items, the ordinal alpha obtained was 70% for the knowledge subscale, 69% for the attitudes and risk perceptions subscale, 76% for the practices subscale, and 77% for the entire questionnaire. Therefore, the questionnaire presented an acceptable internal consistency. Regarding CFA, we analyzed the three subscales of the questionnaire separately assuming that the covariance among items is due to a single common factor. For the knowledge subscale, the explained variance was 9.6%. For the attitudes and risk perceptions subscale, the explained variance was 19.3%. For the practices subscale, the explained variance was 14.4%. Fully details of the internal consistency analysis and of the CFA are in Supplementary Material.

The mean knowledge subscale score was 11.31 ± 2.89, while the mean score for the practices subscale was 47.62 ± 6.60. This means that the participants answered 0.8% of the knowledge questions and 41% of the practice questions appropriately. Only 14.7% were aware of the common symptoms of COVID-19 and only 0.3% were fully aware of the warning signs that require a referral to a hospital. The knowledge and practices subscale had a correlation of 0.33 (*p* < 0.001).

The most frequent correct response from the knowledge subscale questions was about the possibility of getting COVID-19 and being asymptomatic (93.2%) and the least frequent one was about the proper disposal of a used face mask (5.9%). In terms of attitudes and risk perceptions, 16.7% believed that preventive measures were highly observed in the community, while 67.2% believed that they themselves very much used preventive measures. Moreover, 5.6% considered themselves at high risk for COVID-19 and 37% considered it a serious illness. This disease had negative effects on the routine activities of most of the participants (87.9%). The most frequent appropriate practice was using a face mask (83.1%), and the least frequent one was about keeping social distance (9.6%). Furthermore, 54.5% of the participants had increased their water intake, 13% traveled outside the city, and 18.9% were in contact with patients with at least one of the three symptoms of fever, dry cough, or dyspnea during 2 weeks before this study. It was found that 21.5% declared that their first response was to refer to the medical clinics upon the occurrence of any suspected symptom of COVID-19.

Regarding the relationship between the subscales of the questionnaire and the sociodemographic variables, univariate and multivariate analyses were carried out. The univariable analysis (Table 2) showed that: males had a lower level of correct knowledge about COVID-19 than females (*t* = 3.95, *p* < 0.001); adolescents living in cities had a higher level of correct knowledge about COVID-19 than those living in smaller towns (*t* = 4.92, *p* < 0.001); and adolescents that had a member of their family receiving financial aid had a lower level of correct knowledge about COVID-19 and scored less in attitudes and risk perceptions than those who did not have this type of family member (*t* = −4.24, *p* < 0.001 and *t* = −2.08, *p* = 0.042, respectively).

**TABLE 2:** Univariable analysis.

Variables	Knowledge			Practices			Attitudes and risk perceptions		
			
	Mean (SD)	Statistic	*p*	Mean (±SD)	Statistic	*p*	Mean (±SD)	Statistic	*p*
**Age**									
Early (up to 14 y/o)	23.46 (2.32)	*F* = 0.03	0.968	39.14 (4.25)	*F* = 0.84	0.430	51.91 (6.31)	*F* = 3.40	0.034
Middle (between 15 and 17 y/o)	23.43 (2.63)			38.41 (5.14)			49.74 (7.42)		
Late (18 or more y/o)	23.58 (2.23)			39.12 (5.25)			51.06 (5.65)		
**Gender**									
Male	22.79 (2.71)	*t* = −3.95	<0.001	49.65 (7.55)	*t* = −1.55	0.122	38.04 (5.51)	*t* = −1.92	0.056
Female	23.87 (2.25)			50.89 (6.80)			39.06 (4.24)		
**Place of residence**									
City (more than 10,000 inhabitants)	23.95 (2.20)	*t* = 4.92	<0.001	50.60 (6.92)	*t* = 0.64	0.519	38.72 (4.51)	*t* = 0.34	0.734
Town (less than 10,000 inhabitants)	22.50 (2.83)			50.09 (7.45)			38.53 (5.61)		
**Level of education**									
None	19.50 (2.12)	*F* = 3.20	0.023	50.50 (12.02)	*F* = 0.42	0.741	31.50 (2.12)	*F* = 1.67	0.172
Primary school	23.15 (2.71)			50.83 (7.14)			38.47 (5.33)		
Secondary school	23.67 (2.33)			49.96 (7.34)			38.89 (4.54)		
Higher secondary	24 (2)			50.60 (4.68)			39.15 (4.59)		
**Sentimental relationship**									
No	23.55 (2.36)	*t* = 1.65	0.105	50.49 (7.01)	*t* = 0.44	0.654	38.67 (4.38)	*t* = 0.15	0.882
Yes	22.68 (3.40)			49.97 (7.73)			38.50 (7.73)		
**Financial aid**									
Yes	21.85 (3.19)	*t* = −4.24	<0.001	48.89 (8.56)	*t* = −1.49	0.136	36.58 (7.47)	*t* = −2.08	0.042
No	23.89 (2.25)			50.57 (6.88)			38.89 (4.27)		

*t-Test for categories with two variables and analysis of variance (ANOVA) for the rest of the categories.*

Adolescents who had never repeated a year in school scored higher in knowledge compared to those who had repeated one or more school years (*F* = 20.48, *p* < 0.001). Moreover, adolescents who had never repeated a year in school scored higher in attitudes and risk perceptions comparing to those who had repeated two or more years (*F* = 5.35, *p* = 0.005). There were no differences in terms of whether the participant was in a sentimental relationship, the number of siblings they had, whether they were currently studying, or their level of education.

According to the multivariate analysis, knowledge about COVID-19 was higher in women, and in those with a higher level of education; and was lower in those who lived in smaller towns, and in those who had a member of their family receiving financial aid (Table 3). Concerning practices, no significant variables were obtained (Table 4). Regarding attitudes and risk perceptions, younger adolescents had a higher score, and those who had a member of their family receiving financial aid had a lower score (Table 5). No multicollinearity was reported.

**TABLE 3:** Linear regression analysis of the demographic determinants of adolescents’ “knowledge” toward COVID-19.

*R*^2^ adjusted = 0.156	*F*(6,303) = 10.522				*p* < 0.001	
		
Model	Unstandardized coefficients		Standardized coefficients	*t*	*p*	95% CI for *B*
		
	*B*	SE	Beta			
(Constant)	22.013	1.796		12.255	0.000	[18.478, 25.547]
Gender	0.915	0.278	0.174	3.294	0.001	[0.369, 1.462]
Age	–0.144	0.133	–0.089	–1.089	0.277	[−0.405, 0.117]
Sentimental relationship	–0.750	0.418	–0.098	–1.794	0.074	[−1.571, 0.072]
Place of residence	−**0.945**	0.282	–0.181	–3.349	**0.001**	[−1.501, −0.390]
Level of education	**0.727**	0.336	0.177	2.165	**0.031**	[0.066, 1.388]
Financial aid	**1.353**	0.378	0.195	3.579	**0.000**	[0.609, 2.097]

*Dependent variable: Knowledge. Significant differences (p ≤ 0.05) are highlighted in bold font.*

*CI, confidence interval.*

**TABLE 4:** Linear regression analysis of the demographic determinants of adolescents’ “practices” toward COVID-19.

*R*^2^ adjusted = 0.003	*F*(6,303) = 1.144				*p* < 0.337	
		
Model	Unstandardized coefficients		Standardized coefficients	*t*	*p*	95% CI for *B*
		
	*B*	SE	Beta			
(Constant)	53.557	5.619		9.531	0.000	[42.499, 64.614]
Gender	1.179	0.869	0.078	1.356	0.176	[−0.532, 2.889]
Age	–0.485	0.415	–0.104	–1.169	0.243	[−1.302, 0.331]
Sentimental relationship	–0.312	1.307	–0.014	–0.239	0.811	[−2.884, 2.259]
Place of residence	–0.410	0.883	–0.027	–0.464	0.643	[−2.148, 1.328]
Level of education	0.315	1.051	0.027	0.300	0.764	[−1.752, 2.383]
Financial aid	1.288	1.183	0.065	1.089	0.277	[−1.040, 3.616]

*Dependent variable: practices.*

*CI, confidence interval.*

**TABLE 5:** Linear regression analysis of the demographic determinants of adolescents’ “attitudes and risk perceptions” toward COVID-19.

*R*^2^ adjusted = 0.035	*F*(6,303) = 2.869				*p* < 0.010	
		
Model	Unstandardized coefficients		Standardized coefficients	*t*	*p*	95% CI for *B*
		
	*B*	*SE*	Beta			
(Constant)	39.060	3.685		10.599	0.000	[31.808, 46.312]
Gender	0.720	0.570	0.071	1.263	0.208	[−0.402, 1.841]
Age	−**0.543**	0.272	–0.175	–1.994	**0.047**	[−1.078, −0.007]
Sentimental relationship	0.296	0.857	0.020	0.345	0.730	[−1.390, 1.982]
Place of residence	–0.361	0.579	–0.036	–0.624	0.533	[−1.501, 0.778]
Level of education	1.204	0.689	0.153	1.748	0.082	[−0.152, 2.560]
Financial aid	**2.038**	0.776	0.153	2.626	**0.009**	[0.511, 3.564]

*Dependent variable: attitudes and risk perceptions. Significant differences (p ≤ 0.05) are highlighted in bold font.*

*CI, confidence interval.*

## Discussion

In adolescence, the personal and social aspects of the individual are redefined through processes of exploration, differentiation of the family environment, and the search for belonging and meaning of life (Krauskopof, 1999). Risk perception is the subjective judgment that people make regarding the characteristics, severity, and way in which the risk is managed (Cori et al., 2020). According to the specific population group, risk perception will differ. In adolescents, everything unknown encourages them to explore since they are at a stage of discovery, and the less knowledge they have about something, the greater the degree of curiosity, especially if they are stimulated by their peers (Acero González et al., 2007). In adolescence, gradual exposure to risks is part of the growth of the individual, and this allows for the development of skills and the strengthening of resources (Peñaherrera Sánchez, 1998).

The lack of evaluation methods that allow for the study of the perception of risk in adolescents in the context of COVID-19 has produced little previous evidence. However, the literature regarding risk perception in other risk situations such as alcohol and drug use (Herruzo et al., 2016), and HIV infection (Navarro Lechuga and Vargas Morath, 2005) is extensive. The present study has made it possible to delve into the existing relationships between the knowledge, the attitudes, the risk perceptions, and the practices toward COVID-19 of adolescents. To the best of our knowledge, this is the first study to examine the psychometric properties of the knowledge, attitudes, risk perceptions, and practices toward the COVID-19 pandemic questionnaire in Spanish adolescents aged 12–18 years.

Regarding the differences caused by age, it has been observed that adolescents between 15 and 17 years of age are those who present a lower risk perception. This could be due to the fact that it is at this age when the family is no longer the privileged space to confirm abilities and self-esteem, producing a differentiation from the family group in the search for autonomy and in the exploration of personal capacities; placing themselves in front of the world and questioning previous behaviors and positions (Krauskopof, 1999). The results show that female adolescents present greater knowledge regarding COVID-19 than male adolescents, presenting a tendency to have a greater risk perception of the disease. Most of our sample was female, and according to a recent study, sex was the largest predictor of stress, which may have enhanced the possibility that women would embrace COVID-19 prevention instructions and seek knowledge (Mondal et al., 2021). Moreover, previous studies reflect gender differences regarding risk perception in men and women, with men presenting the most reckless practices (Alvarado et al., 2011; Giménez-García et al., 2016; Herruzo et al., 2016). Likewise, adolescents who reside in cities present greater knowledge about COVID-19, compared to those who reside in towns, which could be due to greater access to information and education in cities (Eccles, 2013). This is an aspect that coincides with the fact that more educated adolescents present a higher level of knowledge of COVID-19 (Honarvar et al., 2020). In addition, those adolescents who have someone in their family receiving financial aid (a sign of low economic status) also have less knowledge and less risk perception (Oliva et al., 2011). The higher level of education in adolescents residing in cities, and in those with a better economic status, was an expected result. There is accumulated evidence that indicates that the level of education tends to be higher in families with better socioeconomic status as well as in families that have parents with a higher level of education, and also among those who reside in cities (Oliva et al., 2011; Eccles, 2013; Honarvar et al., 2020).

Regarding the different subscales of the questionnaire, it has been observed how the knowledge subscale regarding COVID-19 is explained by the age of the adolescent, their place of residence, their level of education, and whether someone in their family is receiving financial aid. The adolescent’s gender is no longer significant, due to the strong relationship of the other sociodemographic variables. As previously stated, these results are consistent with previous studies (Oliva et al., 2011; Eccles, 2013; Honarvar et al., 2020). In addition, the increase in age allows for greater cognitive development in the adolescent that leads to the possibility of improving their learning and knowledge.

The practices subscale does not present a significant relationship with any variable, which could be due to the characteristics of the questionnaire such as the use of dichotomous answers (i.e., yes/no), since the answers are very restricted. It would be more appropriate if they had all been Likert-type or had more response options. Furthermore, adolescents are immersed in an evolutionary period where risk is perceived differently. They tend to think that their actions are not going to have any danger in the future despite appreciating that their parents or their close adult relatives try to prevent them from such behaviors (Ballester Arnal et al., 2000). In addition, not considering the sources of information about COVID-19, could be biasing the results regarding preventive practices, since both adolescents and adults (mainly young adults) have obtained the information about COVID-19 through social networks, which favor the transmission of erroneous or misleading information (Salamero et al., 2021). This fact has a direct impact on knowledge about the disease and its preventive measures, which is associated with the change in risk behaviors (Rosabal et al., 2016).

The attitudes and risk perceptions subscale is explained by the age of the adolescent and whether someone in their family is receiving financial aid. This latter aspect coincides with the fact that the individual risk factors of adolescents include belonging to a family with lower income (Valenzuela Mujica et al., 2013). In addition, it has been confirmed that risk perception in the context of COVID-19 is related to age, which is an aspect that coincides with other studies in which as one advances in age, there is a greater permissiveness in the face of risk (de la Villa Moral and Ovejero Bernal, 2005). At the beginning of adolescence, there is a strong self-awareness of the needs and desires of the elderly to understand and support. However, in middle adolescence, there is a search for autonomy, a differentiation from the family group, an exploration of personal capacities, and a questioning of previous experience. These enable the adolescent to develop an internal locus of control and create identity, with there now being a greater capacity for self-care and mutual care in this stage (Krauskopof, 1999).

In general, the risk perception increases significantly with age. However, this is not always the case, since other variables are involved in this process (that is, intuitive and automatic feelings, false beliefs of superiority, attempts to minimize negative consequences, etc.), which make the progression non-linear, although life is in danger (Del Castillo, 2012; Stanojlovic, 2015). Regarding the risk perception in adults, in studies carried out on other infectious diseases, they found a significant statistical association between low knowledge about the disease and low risk perception of infection (Leveaú Laulate et al., 2011; Araujo López et al., 2013). Specifically, in the face of COVID-19, the few existing studies affirm that adults have an inadequate risk perception (González Gacel et al., 2021), but the elderly do seem to present a high risk perception, due to mortality from disease in their age group (Fernández-Ballesteros et al., 2020).

In any case, we must keep in mind that preventive behavior is complicated and multifaceted. A recent study comparing Indian and American populations discovered that, despite having less knowledge, Indians adhered to preventative practices better (Sinharoy et al., 2021). As a result, the link between knowledge and preventive behavior should be thoroughly investigated.

Knowing what adolescents know, and what their practices and attitudes are regarding COVID-19 is a valuable source of information for the design of educational, preventive, and communicative strategies. Previous studies confirm that identifying and increasing the level of knowledge about the disease could reduce risk behaviors since it is a central aspect that influences the risk perception of adolescents (DiClemente et al., 2002; Navarro Lechuga and Vargas Morath, 2005).

### Limitations

The findings of this research have several limitations that should be taken into consideration for future investigations. First, as we used non-probabilistic snowball sampling, it would be interesting to use a random sampling method in order to generalize the outcomes. Moreover, the instrument’s stability over time was not analyzed due to the fact that the study was cross-sectional. Thus, it would be useful to carry out longitudinal research projects to examine if the results are maintained throughout weeks. In addition, more research is needed to determine the factor structure of the questionnaire. Finally, future research might consider include certain vaccination-related questions, given the vaccine had not yet been developed at the time the questionnaire was created.

### Strengths

Despite these limitations, our results have some implications that may be significant. Firstly, the validation of the questionnaire permits researchers to go more in-depth in the study of the knowledge, practices, attitudes, and risk perceptions toward COVID-19 in adolescents. Thus, strategies could be implemented to develop better knowledge, attitudes, and practices when health and social crises occur. Moreover, as the results provide information on how adolescents behave, this serves to design public and preventive policies that fit the behaviors of adolescents. This would, therefore, favor the creation of more effective interventions, both in the educational and clinical fields.

## Conclusion

The knowledge, attitudes, risk perceptions, and practices of adolescents toward the COVID-19 pandemic questionnaire has proven to be a reliable tool in the Spanish adolescent population after some modifications. Regarding the results of the questionnaire, the knowledge of Spanish adolescents about COVID-19 is quite low. On the other hand, as far as practices are concerned, they show great appropriateness. Finally, attitudes and risk perceptions were influenced by age and financial aid.

## Data Availability Statement

The raw data supporting the conclusions of this article will be made available by the authors, without undue reservation.

## Ethics Statement

All procedures performed in studies involving human participants were in accordance with the ethical standards of the institutional and/or national research committee (Clinical Research Ethics Committee of Aragón – CEICA, PI20/472) and with the 1964 Helsinki declaration and its later amendments or comparable ethical standards. Informed consent was obtained from all individual participants included in the study. Written informed consent to participate in this study was provided by the participants’ legal guardian/next of kin.

## Author Contributions

ÁA-M, AA-L, and OG-S led the design, developed the study and had the original idea, coordinated the fieldwork, and undertook the fieldwork. ÁA-M, AA-L, OG-S, and BO-B wrote the first draft of the manuscript. All authors contributed to the article and approved the submitted version.

## Conflict of Interest

The authors declare that the research was conducted in the absence of any commercial or financial relationships that could be construed as a potential conflict of interest.

## Publisher’s Note

All claims expressed in this article are solely those of the authors and do not necessarily represent those of their affiliated organizations, or those of the publisher, the editors and the reviewers. Any product that may be evaluated in this article, or claim that may be made by its manufacturer, is not guaranteed or endorsed by the publisher.

## Footnotes

1
https://www.facebook.com

2
http://instragram.com

3
https://twitter.com
