# Supplementary material for: Knowledge, Attitudes, Risk Perceptions, and Practices of Spanish Adolescents Toward the COVID-19 Pandemic: Validation and Results of the Spanish Version of the Questionnaire
Source: Front Psychol. 2022 Jan 4;12:804531. doi: 10.3389/fpsyg.2021.804531 (PMC8763840; doi:10.3389/fpsyg.2021.804531)
Supplement: Supplementary file 1 [file Data_Sheet_1.PDF]

# IMPACTO Y PERCEPCIÓN DEL RIESGO ANTE LA PANDEMIA CAUSADA POR EL COVID-19 EN ADOLESCENTES ESPAÑOLES

Nos dirigimos a ti para solicitar tu participación en un proyecto de investigación que estamos realizando en la Universidad de Zaragoza. Tu participación es voluntaria, pero es importante para obtener el conocimiento que necesitamos. Este proyecto ha sido aprobado por el Comité de Ética de la Investigación de la Comunidad Autónoma de Aragón (CEICA) (C.P. - C.I. PI20/472), pero antes de tomar una decisión es necesario que leas y entiendas esta información. Puedes realizar todas las preguntas que consideres necesarias al email acasensi@unizar.es

Si tienes 13 años o menos, por favor, avisa a tu padre, madre o tutor legal para que pueda leer esta información contigo, además tendrán que autorizar que puedas completar esta encuesta.

Se solicita tu colaboración porque perteneces la población con una edad comprendida entre 12 y 18 años. En total en el estudio participarán 253 participantes de esa misma edad.

Con este estudio queremos analizar la percepción que tienen los adolescentes de la pandemia causada por la COVID-19, el conocimiento sobre la misma y el riesgo que creéis que conlleva. Dada la situación actual que hemos vivido todos nosotros creemos que puede ayudar a comprender cómo gestionan los adolescentes estas situaciones excepcionales. Esperamos que ayude también a entender mejor a los adolescentes en este tipo de situaciones, ya que no son del todo comprendidos por la sociedad en general.

Si decides participar solo tendrás que rellenar, una vez, la siguiente encuesta online y anónima. Te llevará unos 15 minutos. La encuesta se va a realizar a través de la plataforma Google Forms, por lo que tendrás que revisar y aceptar su política de privacidad. Vamos a llevar a cabo esta encuesta durante los meses de octubre, noviembre y diciembre de 2020 y enero de 2021.

Participar en esta encuesta no supone ningún riesgo para ti, ya que todos los datos van a ser anónimos, no se va preguntar ningún dato que permita identificar quién ha respondido. Además, eres libre de parar la realización de la encuesta en cualquier momento, así como no contestar a aquellas preguntas que no quieras. Aunque para el éxito del proyecto necesitamos que se contesten todas, sino la gran mayoría, de las preguntas.

Al tratarse de un estudio de investigación orientado a generar conocimiento, no es probable que obtengas ningún beneficio por tu participación si bien contribuirás al avance científico y al beneficio social de los adolescentes.

Respecto a tus datos personales, la investigadora principal de este proyecto, Ángela Asensio, es responsable de los mismos. Tus datos personales serán tratados exclusivamente para el trabajo de investigación a los que hace referencia este documento. El tratamiento de los datos de este estudio queda legitimado por su consentimiento a participar. No se cederán datos a terceros salvo obligación legal. Podrás ejercer tus derechos de acceso, rectificación, supresión y portabilidad de sus datos, de limitación y oposición a su tratamiento, de conformidad con lo dispuesto en el Reglamento General de Protección de Datos (RGPD 2016/679) ante el investigador principal del proyecto, pudiendo obtener información al respecto escribiendo un correo electrónico a la dirección acasensi@unizar.es

*Obligatorio

1. Antes de comenzar la encuesta, indica que tienes 12 años o más y que aceptas la política de privacidad de Google Forms: https://policies.google.com/privacy. *

*Selecciona todos los que correspondan.*

- [ ] Sí, tengo 12 años o más. He leído y acepto la política de privacidad de Google Forms.

## Consentimiento Informado para MAYORES DE 13 AÑOS

Tienes que marcar las opciones siguientes sólo si tienes más de 13 años. Si tienes 13 años o menos, selecciona la casilla correspondiente y ve al siguiente apartado.

2. Marca las opciones que correspondan: *

*Selecciona todos los que correspondan.*

- [ ] Tengo 13 años o menos (Marca esta opción y ve al siguiente apartado).
- [ ] He leído la información del estudio previamente mostrada.
- [ ] He podido hacer preguntas sobre el estudio y he recibido suficiente información sobre el mismo, en caso de haberlas realizado.
- [ ] Comprendo que mi participación es voluntaria.
- [ ] Comprendo que puedo retirarme del estudio cuando quiera y sin tener que dar explicaciones.
- [ ] Presto libremente mi consentimiento para participar en este estudio y doy mi consentimiento para el acceso y utilización de mis datos conforme se estipula en la información del estudio previamente mostrada.
- [ ] Deseo ser informado/a sobre los resultados del estudio. De ser así deberás proporcionar un email de contacto (a continuación, donde pone "otro"):

Otro: [ ] ______________________

## Consentimiento Informado para 13 AÑOS O MENOS

Si tienes 13 años o menos, por favor, avisa a tu padre, madre o tutor legal para que pueda leer esta información contigo.

3. Si tienes 13 años o menos, tu padre, madre o tutor legal deberá reflejar a continuación el año de su nacimiento. Si tienes más de 13 años, escribe tu propia fecha de nacimiento. *

______________________

4. Tu padre, madre o tutor legal deberá marcar las opciones que correspondan: *

*Selecciona todos los que correspondan.*

- [ ] Tengo más de 13 años (ya he completado el apartado anterior).
- [ ] He leído la información del estudio previamente mostrada.
- [ ] He podido hacer preguntas sobre el estudio y he recibido suficiente información sobre el mismo, en caso de haberlas realizado.
- [ ] Comprendo que la participación de mi hijo/a es voluntaria.
- [ ] Comprendo que puedo retirarlo/la del estudio cuando quiera y sin tener que dar explicaciones.
- [ ] Presto libremente mi consentimiento para que mi hijo/a participe en este estudio y doy mi consentimiento para el acceso y utilización de sus datos conforme se estipula en la información del estudio previamente mostrada.
- [ ] Deseo ser informado/a sobre los resultados del estudio. De ser así deberás proporcionar un email de contacto (a continuación, donde pone "otro"):

Otro: [ ] ______________________

## Cuestionario Datos Sociodemográficos

5. Género *

*Marca solo un óvalo.*

- ( ) Hombre
- ( ) Mujer
- ( ) Otro: ______________________

6. Edad *

______________________

7. ¿Cuál es tu país de nacimiento?: *

*Marca solo un óvalo.*

- ( ) España
- ( ) Otro: ______________________

8. Estado Civil *

*Marca solo un óvalo.*

- Soltero/a
- Casado/a
- En pareja
- Divorciado/a
- Otro: ____________________

9. ¿Dónde vives? *

*Marca solo un óvalo.*

- Ciudad (más de 10.000 habitantes)
- Pueblo (menos de 10.000 habitantes)

10. ¿Estás estudiando? *

*Marca solo un óvalo.*

- Sí
- No
- No sé/No contesto

11. ¿Qué nivel de estudios has completado? *

*Marca solo un óvalo.*

- Ninguno
- Ciclo Primaria
- ESO-Secundarios
- Bachillerato
- Formación Profesional Reglada
- Talleres Ocupacionales
- Escuela de Adultos cursando certificado de ESO (>16años)
- Escuelas de Oficios
- Talleres propios de centros o Fundaciones/ONG's (cursos de idiomas, etc.)
- No sé/No contesto
- Otro: ____________________

12. ¿Has repetido curso alguna vez? *

*Marca solo un óvalo.*

- No
- Sí, un curso
- Sí, dos o más cursos
- No sé/No contesto

13. Actualmente, ¿alguna de las personas con las que vives recibe ayuda económica? *

*Marca solo un óvalo.*

- Sí
- No
- No sé/No contesto

Cuestionario sobre los conocimientos, las actitudes, la percepción del riesgo y las prácticas de los adolescentes hacia la pandemia por la COVID-19

**Conocimientos sobre la COVID-19. Nos gustaría conocer tus conocimientos sobre la COVID-19. Para ello, contesta a cada una de las preguntas. Puedes contestar "no lo sé".**

14. K1) ¿Es posible que alguien contraiga COVID-19 si es asintomático? *

15. K2) Después de infectarse con COVID-19, ¿cuántos días se tarda en mostrar sus síntomas? *

16. K3) ¿Cuáles son las vías de transmisión de COVID-19? *

17. K4) ¿Cuáles son las vías preventivas de infección por COVID-19? *

18. K5) ¿Qué síntomas de COVID-19 son más frecuentes? *

19. K6) En términos de COVID-19, ¿cuáles son las señales de advertencia para ir al hospital? *

20. K7) ¿A qué número de teléfono llama si tiene alguna pregunta sobre COVID-19? *

21. K8) ¿Qué grupos de personas tienen un mayor riesgo de mortalidad después de contraer COVID-19? *

22. K9) ¿Cómo se deben lavar las manos correctamente? *

23. K10) ¿Cuál es el tiempo mínimo para lavarse las manos correctamente? *

24. K11) ¿Conoce los diferentes tipos de mascarillas faciales y sus usos? *

25. K12) ¿Cuánto tiempo puedes dejar una mascarilla en tu rostro? *

26. K13) ¿Cómo se debe usar correctamente una mascarilla? *

27. K14) ¿Cómo se debe desechar correctamente una mascarilla usada? *

28. K15) ¿Cómo se debe desechar correctamente un guante usado? *

29. K16) ¿Cómo se debe cuidar a los pacientes con COVID-19 en casa? *

30. K17) ¿La vacuna contra la gripe protege contra COVID-19? *

Cuestionario sobre los conocimientos, las actitudes, la percepción del riesgo y las prácticas de los adolescentes hacia la pandemia por la COVID-19

31. Actitudes y percepción del riesgo sobre la COVID-19. Marca una respuesta para cada una de las preguntas que te presentamos, marcando la casilla que corresponde a la opción con la que mejor te identificas. Recuerda no hay respuestas ni buenas ni malas. *

*Marca solo un óvalo por fila.*

| | Mucho | Bastante | Algo | Poco | Nada |
|---|---|---|---|---|---|
| A1 ¿En qué medida se observan en la comunidad las medidas preventivas contra COVID-19? | ⬭ | ⬭ | ⬭ | ⬭ | ⬭ |
| A2 ¿En general, en qué medida observas las medidas preventivas contra COVID-19? | ⬭ | ⬭ | ⬭ | ⬭ | ⬭ |
| A3 ¿En qué grado crees que podrías contraer COVID-19? | ⬭ | ⬭ | ⬭ | ⬭ | ⬭ |
| A4 ¿Cuánto temes contraer COVID-19? | ⬭ | ⬭ | ⬭ | ⬭ | ⬭ |
| A5 ¿Cuánto temes que tu familia te contagie el COVID-19? | ⬭ | ⬭ | ⬭ | ⬭ | ⬭ |
| A6 ¿Cuánto les preocupa a los miembros de tu familia contraer COVID-19? | ⬭ | ⬭ | ⬭ | ⬭ | ⬭ |
| A7 ¿Hasta qué punto crees que COVID-19 es una enfermedad peligrosa y mortal? | ⬭ | ⬭ | ⬭ | ⬭ | ⬭ |
| A8 Si sospechas que tienes COVID-19, ¿Cómo de preparado estás para realizarte el test? | ⬭ | ⬭ | ⬭ | ⬭ | ⬭ |
| A9 Si se sospecha que tienes COVID-19, ¿en qué medida te haces responsable de la salud de los demás y sigues las medidas preventivas con mayor precisión? | ⬭ | ⬭ | ⬭ | ⬭ | ⬭ |
| A10 ¿Hasta qué punto ha tenido el COVID-19 una influencia negativa en tu vida cotidiana? | ⬭ | ⬭ | ⬭ | ⬭ | ⬭ |

32. Prácticas de los participantes sobre la COVID-19. Marca una respuesta para cada una de las preguntas que te presentamos, marcando la casilla que corresponde a la opción con la que mejor te identificas. Recuerda no hay respuestas ni buenas ni malas. *

*Marca solo un óvalo por fila.*

| | Siempre | A menudo | A veces | Pocas veces | Nunca |
|---|---|---|---|---|---|
| P1 ¿Con qué frecuencia te lavas o desinfectas las manos? | ◯ | ◯ | ◯ | ◯ | ◯ |
| P2 ¿Con qué frecuencia evitas tocarte la cara y los ojos con las manos sin lavar? | ◯ | ◯ | ◯ | ◯ | ◯ |
| P3 ¿Con qué frecuencia usas una mascarilla facial? | ◯ | ◯ | ◯ | ◯ | ◯ |
| P4 ¿Con qué frecuencia usas una mascarilla, un pañuelo de papel o un codo al estornudar, toser o moquear la nariz? | ◯ | ◯ | ◯ | ◯ | ◯ |
| P5 ¿Con qué frecuencia tiras las mascarillas y los pañuelos de papel usados en bolsas separadas y luego en la papelera? | ◯ | ◯ | ◯ | ◯ | ◯ |
| P6 ¿Con qué frecuencia desinfectas regularmente las superficies de los objetos y lugares personales? | ◯ | ◯ | ◯ | ◯ | ◯ |
| P7 ¿Con qué frecuencia le das la mano a los demás? | ◯ | ◯ | ◯ | ◯ | ◯ |
| P8 ¿Con qué frecuencia besas a los demás? | ◯ | ◯ | ◯ | ◯ | ◯ |
| P9 ¿Con qué frecuencia abrazas a los demás? | ◯ | ◯ | ◯ | ◯ | ◯ |
| P10 ¿Con qué frecuencia te mantienes al menos entre 1m y 1,5 m de distancia de los demás? | ◯ | ◯ | ◯ | ◯ | ◯ |
| P11 ¿Con qué frecuencia sigues las noticias sobre COVID-19? | ◯ | ◯ | ◯ | ◯ | ◯ |
| P12 ¿Con qué frecuencia tratas de enseñar a otras personas sobre la prevención del COVID-19? | ◯ | ◯ | ◯ | ◯ | ◯ |

| | | | | | |
|---|---|---|---|---|---|
| P13 ¿Con qué frecuencia tú o tu familia cocinan correctamente los huevos o la carne antes de consumirlos? | ◯ | ◯ | ◯ | ◯ | ◯ |

33. P 14 ¿Estuviste en contacto con personas con al menos uno de los tres síntomas de fiebre, tos seca o dificultad para respirar durante las 2 semanas previas a este estudio? *

*Marca solo un óvalo.*

◯ Sí

◯ No

34. P 15 ¿Viajaste fuera de la ciudad durante las 2 semanas previas a este estudio? *

*Marca solo un óvalo.*

◯ Sí

◯ No

35. P16 ¿Has aumentado tu consumo de agua? *

*Marca solo un óvalo.*

◯ Sí

◯ No

36. P 17 ¿Has disminuido los desplazamientos innecesarios fuera del hogar? *

*Marca solo un óvalo.*

◯ Sí

◯ No

37. P 18 ¿Qué es lo primero que harías si notaras la aparición de cualquier síntoma sospechoso de la COVID-19? *

Este contenido no ha sido creado ni aprobado por Google.

Google Formularios
