# Supplementary material for: Knowledge, Attitudes, Risk Perceptions, and Practices of Spanish Adolescents Toward the COVID-19 Pandemic: Validation and Results of the Spanish Version of the Questionnaire
Source: Front Psychol. 2022 Jan 4;12:804531. doi: 10.3389/fpsyg.2021.804531 (PMC8763840; doi:10.3389/fpsyg.2021.804531)
Supplement: Supplementary file 2 [file Data_Sheet_2.docx]

**Supplementary material 2**

**The internal consistency analysis indicated that a few items with low or negative correlation needed to be eliminated: items 10, 7, and 11 of the knowledge subscale; and items 14, 15, and 16 of the practices subscale. After deleting those items, the ordinal alpha obtained was 74% for the knowledge subscale, 69% for the attitudes and risk perceptions subscale, 82% for the practices subscale, and 81% for the entire questionnaire.**

**Regarding the Confirmatory Factors Analysis (CFA), we analyzed the three subscales of the questionnaire separately: knowledge, attitudes and risk perceptions, and practices. For the knowledge subscale,** the KMO test obtained a value of 0.69, and the Bartlett sphericity test obtained a statistically significant value (p < 0.001). A dimensional matrix of 14 items was extracted, as well as one factor explaining 9.6% of the variance. **For the attitudes and risk perceptions subscale,** the KMO test obtained a value of 0.71, and the Bartlett sphericity test obtained a statistically significant value (p < 0.001). A dimensional matrix of 10 items was extracted, as well as one factor explaining 19.3% of the variance**. For the practices subscale,** the KMO test obtained a value of 0.77, and the Bartlett sphericity test obtained a statistically significant value (p < 0.001)**. A** dimensional matrix of 15 items was extracted, as well as one factor explaining 14.4% of the variance.
